# Examination of Post-Traumatic Stress Reaction, Quality of Life, and Loneliness Levels in Adolescents Who Experienced an Earthquake

**DOI:** 10.1007/s40653-026-00822-5

**Published:** 2026-02-09

**Authors:** Gamze Akay, Elif Simay Koç, Hatice Oğuzhan

**Affiliations:** 1 Faculty of Health Sciences, Department of Child Health and Disease Nursing, Artvin Coruh University, Artvin, Turkey; 2 Kilis 7 Aralık University Vocational School of Health Services Kilis, Kilis, Turkey; 3 Gümüşhane University Vocational School of Health Services Gümüşhane, Gümüşhane, Turkey

**Keywords:** Adolescent, Earthquake, Trauma, Loneliness, Quality of life

## Abstract

The aim of this study is to investigate the relationship between post-traumatic stress, quality of life, and loneliness levels in adolescents following the earthquakes centered in Kahramanmaraş. The research, designed as descriptive and correlational, was conducted with 701 children aged 13–18 who experienced the earthquake in Kahramanmaraş. Data were collected through a Demographic Information Form, the Child Post-Traumatic Stress Reaction Index, the Pediatric Quality of Life Inventory, and the UCLA Loneliness Scale-Short Form. Ethical principles were adhered to throughout the study. Female adolescents were found to have higher levels of post-traumatic stress (*t* = 3.508, *p* < 0.001) and lower quality of life (*t* = 4.223, *p* < 0.001). The adolescents exhibited severe levels of post-traumatic stress (55.25 ± 13.80). It was determined that the mother’s education level (*F* = 4.423, *p* = 0.002), the father’s occupation (*F* = 2.859, *p* = 0.036), and perceived income level (*F* = 5.912, *p* = 0.003) significantly influenced adolescents’ loneliness levels. The father’s occupation (*F* = 3.403, *p* = 0.017) was found to have a significant effect on post-traumatic stress levels, and perceived income level (*F* = 4.753, *p* = 0.009) had a significant effect on quality of life. A moderate negative correlation was found between post-traumatic stress levels and quality of life (*r* = −0.474, *p* < 0.001), and a weak positive correlation was observed between post-traumatic stress and loneliness levels (*r* = 0.215, *p* < 0.001). A weak negative correlation was also found between quality of life and loneliness levels (*r* = −0.284, *p* < 0.001). A moderate negative correlation was found between adolescents’ post-traumatic stress levels and quality of life, while a weak positive correlation was observed between post-traumatic stress levels and loneliness. It is recommended to provide psychological support and guidance services aimed at reducing post-traumatic stress and loneliness levels in adolescents.

## Introduction

Earthquakes are among the most devastating natural disasters, often resulting in substantial loss of life (Olk et al., 2025). Research has demonstrated that such life-threatening experiences can predispose children and adolescents to various psychological disorders (Chen et al., 2023; Düken et al., 2024; İme & Akyıl, 2025; Llach et al., 2025; Yılancıoğlu & Özbaran, 2023). Children’s post-disaster responses differ depending on their developmental stage, and as a result, diverse emotional and behavioral problems may emerge across different age groups (Aral, 2023).

Adolescence is a critical developmental stage characterized not only by hormonal, metabolic, and neurological changes but also by the emergence of behavioral strategies that shape one’s emotional, social, and cultural identity (Mastorci et al., 2024). Adolescents, who are already in the process of adapting to physical, cognitive, and social changes, are particularly vulnerable in the face of disasters (İme & Akyıl, 2025; Kahraman & Çubukcu, 2025). This vulnerability stems from factors such as physical immaturity, dependence on adults, and lack of experience, making adolescents more susceptible to post-earthquake psychological difficulties (Olk et al., 2025). Consequently, given that adolescence is a developmentally sensitive period, traumatic experiences may have a more profound impact (Şerifoğlu & Yilmaz, 2025). Such experiences have been linked to heightened risks of anxiety, depression, social phobia, eating disorders, and post-traumatic stress symptoms (Lasgaard et al., 2011; Laursen & Hartl, 2013; Stickley et al., 2016; Stickley et al., 2024; Düken et al., 2024).

Among the most common psychological outcomes following earthquakes are post-traumatic stress symptoms (PTSS), which typically manifest as re-experiencing, avoidance, negative cognitions, and hyperarousal (Olk et al., 2025; Şerifoğlu & Yilmaz, 2025). The literature consistently indicates that PTSS prevalence among adolescents is high in the aftermath of disasters, symptoms may persist over time, and, if left untreated, they can predispose individuals to lifelong mental health problems (Kalogeratos et al., 2024; Şerifoğlu & Yilmaz, 2025). Moreover, adolescents’ limited ability to verbally express trauma and employ effective coping strategies (Livingston et al., 2025) further exacerbates their risk.

Importantly, the psychological consequences of earthquakes extend beyond psychiatric symptoms and directly affect adolescents’ quality of life (QoL) (Wang et al., 2013; Chou et al., 2004, b). QoL refers to an individual’s subjective evaluation of their physical and mental health, well-being, and living conditions (Çapar & Yelboğa, 2025). Indicators such as health, education, social functioning, infrastructure, and leisure opportunities form the basis of this construct (Hu et al., 2018). Wang et al. (2013) reported that earthquake exposure significantly impaired QoL, particularly in physical, psychological, and environmental domains. Similarly, Chou et al. (2004, b), in their study following the 1999 Taiwan earthquake, found that the severity of psychiatric disorders was inversely associated with QoL scores.

Another frequently observed issue among earthquake-affected populations is loneliness (Mikkelsen et al., 2020; Mikkelsen et al., 2022; Şahin & Göde., 2024). Loneliness may arise either through social exclusion by one’s environment or as a consequence of psychological difficulties leading to social withdrawal (Erözkan, 2009). Although not restricted to any specific age group, loneliness is often experienced more intensely during adolescence (Hu et al., 2025), a period when peer relationships, social support, and a sense of belonging are of critical importance (Aral, 2023). Post-disaster disruptions such as the loss of family members, fragmentation of social networks, and changes in school and living conditions (Aral, 2023) may heighten feelings of loneliness among adolescents.

This study is grounded in the stress-coping model (Lazarus & Folkman, 1984) and relevant trauma theory frameworks, which suggest that individuals’ responses to traumatic events are influenced by their coping resources and social environment. From this perspective, post-traumatic stress symptoms can negatively affect adolescents’ quality of life by impairing emotional, social, and school functioning. Furthermore, these symptoms may exacerbate feelings of loneliness, as affected adolescents may withdraw from social interactions. This theoretical framework justifies the selection of post-traumatic stress, quality of life, and loneliness as key constructs in the present study and provides a rationale for investigating the direction and strength of the relationships among them.

While existing literature has addressed post-traumatic stress and QoL in adolescents after earthquakes, research directly focusing on loneliness in this population remains limited. Moreover, no national or international studies to date have simultaneously examined these three variables together. Yet, evidence suggests that loneliness is interconnected with both QoL (Mikkelsen et al., 2020; Mikkelsen et al., 2022) and post-traumatic stress symptoms (Şahin & Göde., 2024), potentially further undermining adolescents’ psychological well-being.

This study aims to provide a holistic perspective on adolescent mental health in the aftermath of disasters by simultaneously examining post-traumatic stress, quality of life, and loneliness. Furthermore, given that adolescents often tend to underestimate their health problems, which increases vulnerability to both short- and long-term difficulties (Kalogeratos et al., 2024; Mastorci et al., 2024), the significance of this research is further reinforced. Findings are expected to contribute to shaping post-disaster adolescent mental health policies, enhancing awareness among families and educators, and guiding the planning of nursing interventions.

## Methods

### Participants and Procedure

#### Research Type

This research is descriptive and correlational. This research design was chosen to assess the psychological responses of adolescents following the earthquake and to examine the relationships among the variables.

#### Place and Time of the Research

The study was conducted in a district of Kahramanmaraş province, where aftershocks were frequently experienced following the earthquake. Data collection took place between January 1 and January 31, 2025. To ensure that the data collection process was both effective and safe, the study focused on two high schools that were more accessible to the researchers. These two schools were considered suitable sample sources as they represent the overall high school population in the district and include students who experienced the earthquake.

#### Participants and Sample

The population of the study consisted of adolescents aged 13–18 years who experienced the earthquake centered in Kahramanmaraş. From the high schools located in the district where the study was conducted, two schools were selected as clusters for the study. Cluster sampling was chosen due to practical considerations, including accessibility of the schools and the feasibility of conducting face-to-face data collection. While this approach does not provide every individual in the district an equal chance of being selected, it is considered appropriate for representing the student population of the district. Cluster sampling allowed for an efficient and manageable data collection process, while still capturing a diverse range of students who experienced the earthquake (Qureshi et al., 2024). The study included 9th, 10th, 11th, and 12th grade students who experienced the earthquake. The minimum sample size for the study was calculated using the GPOWER method. With a 5% margin of error and a 95% confidence interval, it was determined that a sample of 104 adolescents would be sufficient. However, to increase the power of the study and the quality of the data, the research was completed with a total of 701 adolescents.

### Inclusion Criteria

- Living in Kahramanmaraş,
- Living the February 6 earthquake centered in Kahramanmaraş,
- To be in the 13–18 age group,
- Accepting to participate in the research.

### Exclusion Criteria

- Not wanting to participate in research.

### Measures

Introductory Information Form, Posttraumatic Stress Response Scale for Children, Quality of Life Scale for Children and UCLA Loneliness Scale-Short Form were used to collect the data.

#### Introductory Information Form

An Introductory Information Form was prepared by the researchers to collect descriptive data about the adolescents and their families. The form included a total of 10 questions covering the adolescent’s gender and age, parents’ educational levels, mother’s employment status and occupation if employed, father’s employment status and occupation if employed, perceived family income, and family type.

#### Child Post-Traumatic Stress Reaction Index (PTSD-I)

It is a five-point Likert-type 20-item scale developed by Pynoos et al. (1987) to investigate the severity of PTSD symptoms and adapted into Turkish by Erden et al. A total score between 12 and 24 indicates a mild PTSD reaction, 25–39 indicates a moderate PTSD reaction, 40–59 indicates a severe PTSD reaction and over 60 indicates a very severe PTSD reaction (Erden et al., 1999; Pynoos et al., 1987). Cronbach’s alpha of the scale in our study was 0.860.

#### Pediatric Quality of Life Inventory (PedsQL)

*Pediatric Quality of Life Inventory (PedsQL):* The study used the Turkish version of the Quality of Life Scale for Children (QOLS), developed for adolescents aged 13–18 years and validated for Turkish samples. The scale assesses physical health, social and emotional functioning, as well as school functioning. Each item is linearly reverse-scored on a 0–100 scale, where the responses “almost always = 0,” “often = 1,” “sometimes = 2,” “rarely = 3,” and “never = 4” are converted to 0, 25, 50, 75, and 100 points respectively (0 = 100, 1 = 75, 2 = 50, 3 = 25, 4 = 0). Thus, higher scores obtained from the scale indicate better quality of life. The Cronbach’s alpha coefficient of the scale was reported as 0.93 (Memık et al., 2007; Varni et al., 1999). In our study, the Cronbach’s alpha value of the scale was calculated as 0.888.

#### UCLA Loneliness Scale-Short Form (ULS-8)

The UCLA Loneliness Scale-Short Form originally consists of a single dimension and includes 8 items. However, in the Turkish adaptation study, one of the scale items was removed because it did not exhibit appropriate psychometric properties, and the Turkish form of the scale consisted of 7 items measuring a single dimension. The total score that can be obtained from the four-point Likert-type scale varies between 7 and 28. Higher scores mean that the level of perceived loneliness increases. The Cronbach’s alpha internal consistency coefficient of the scale was reported as 0.74 (Yildiz & Duy, 2014). In this study, Cronbach’s alpha internal consistency value was found to be 0.738.

### Data Collection

The data of the study were collected between January 01–31, 2025 by face-to-face interview technique through school visits. In the introduction part of the questionnaire, an explanation text including the purpose and scope of the research was included for the participants. Participants answered the survey questions after reading and approving this explanation. It took approximately 10–15 minutes for each participant to complete the data tools. It should be noted that the data collection took place nearly two years after the February 6, 2023 earthquake. Therefore, the findings primarily reflect long-term adaptation and chronic psychological outcomes rather than immediate post-disaster responses. In this period, aftershocks and other psychosocial stressors may also have influenced adolescents’ mental health. Moreover, while cluster sampling was chosen for feasibility and safety reasons, the inclusion of only two schools may limit generalizability, as students from these schools may not fully represent all adolescents in the district. Finally, although participation refusal rates were low, the possibility remains that adolescents with more severe distress might have been less willing to participate, which could introduce selection bias.

On the other hand, the use of face-to-face data collection increased response rates and allowed clarification of potential misunderstandings in the questionnaires, thereby strengthening the reliability of the data.

### Data Analysis

The data were analyzed using the Statistical Package for the Social Sciences (SPSS) version 25.0. A significance level of *p* < 0.05 was adopted for all statistical analyses. Mean, standard deviation, frequency and percentage were used to analyze descriptive data. Statistical data were analyzed using independent sample t-test, ANOVA, Pearson Correlation and LSD test. The internal consistency of the scales was evaluated using Cronbach’s alpha coefficient.

### Ethical Principles of Research

Ethical approval (Date: 07.11.2024 No: E.65256) was obtained from the Ethics Committee of XXXXX University and permission was obtained from the relevant institution where the data would be collected. The study was conducted in accordance with the ethical principles of the Declaration of Helsinki. Data were collected on a voluntary basis within the framework of ethical rules. The confidentiality of the participants was prioritized. Participants were not forced to participate in the study. It was clearly stated that the study was not compulsory and participation was completely voluntary. Participants were given a consent form summarizing the procedures, risks and benefits of the study before their participation. They were also assured that they had the right to withdraw from the study without any consequences.

## Findings

### Sociodemographic Characteristics

The mean age of the adolescents was 15.50 ± 1.11 years; 55.2% were female, 32.2% had a mother who graduated from primary school, and 36.9% had a father who graduated from high school. Most mothers were housewives (90.7%), while 45.2% of fathers were self-employed. In terms of socioeconomic status, 64.2% reported income equal to expenses, and 76.3% lived in nuclear families (Table 1).

**Table 1:** Comparison of Adolescents’ Introductory Characteristics with Scale Score Averages (*n* = 701)

Adolescent Age (M ± SD) 15.50 ± 1.11 Min: 13 Max: 18					
	n	%	PTSD-I X ± SD	PedsQL X ± SD	ULS-8 X ± SD
Gender					
Female	387	55.2	56.88 ± 13.93	52.75 ± 14.12	14.64 ± 4.60
Male	314	44.8	53.23 ± 13.40	48.21 ± 14.19	14.13 ± 4.78
Test and p			***t*** **= 3.508** ***p*** **< 0.001**	***t*** **= 4.223** ***p*** **< 0.001**	*t* = 1.416 *p* = 0.155
Mother’s Educational Level					
Illiterate	28	4.0	57.71 ± 11.84	53.28 ± 13.52	16.67 ± 6.03
Primary School	226	32.2	54.50 ± 13.59	51.11 ± 13.02	14.90 ± 4.46
Middle School	211	30.1	56.85 ± 13.35	51.81 ± 14.43	14.50 ± 6.62
High School	186	26.5	54.17 ± 14.37	49.55 ± 15.79	13.42 ± 4.46
University and Above	50	7.1	54.48 ± 15.26	47.18 ± 13.77	14.28 ± 5.27
Test and p			*F* = 1.423 *p* = 0.224	*F* = 1.653 *p* = 0.159	*F* = 4.423 ***p*** **= 0.002**
Father’s Educational Level					
Illiterate	12	1.7	56.83 ± 16.20	52.41 ± 16.14	13.41 ± 4.44
Primary School	128	18.3	55.82 ± 12.72	53.02 ± 14.70	15.29 ± 4.60
Middle School	164	23.4	54.89 ± 12.90	50.71 ± 12.16	14.73 ± 4.70
High School	259	36.9	55.91 ± 14.30	49.96 ± 15.48	13.94 ± 4.62
University and Above	138	19.7	53.76 ± 14.70	49.86 ± 13.88	14.18 ± 4.82
Test and p			*F* = 0.674 *p* = 0.610	*F* = 1.176 *p* = 0.320	*F* = 2.184 *p* = 0.069
Mother’s Occupation					
Housewife	636	90.7	55.28 ± 13.76	50.71 ± 14.26	14.32 ± 4.68
Civil Servant	32	4.6	54.09 ± 14.28	46.65 ± 10.95	14.53 ± 4.15
Worker	33	4.7	55.69 ± 14.50	54.78 ± 17.34	16.03 ± 5.12
Test and p			*F* = 0.132 *p* = 0.877	*F* = 2.630 *p* = 0.073	*F* = 2.083 *p* = 0.125
Father’s Occupation					
Farmer	74	10.6	57.54 ± 14.24	54.32 ± 14.22	15.89 ± 4.71
Civil Servant	189	27.0	52.74 ± 13.02	49.23 ± 13.43	14.08 ± 4.67
Worker	121	17.3	56.84 ± 13.84	50.23 ± 13.65	14.23 ± 4.66
Self-employed	317	45.2	55.61 ± 14.27	50.94 ± 15.00	14.33 ± 4.66
Test and p			*F* = 3.403 ***p*** **= 0.017**	*F* = 2.322 *p* = 0.074	*F* = 2.859 ***p*** **= 0.036**
Perception of Income Status					
Income Less Than Expenses	91	13.0	57.51 ± 13.23	54.02 ± 14.63	15.21 ± 4.52
Income Equal to Expenses	450	64.2	55.19 ± 13.70	50.90 ± 14.31	14.62 ± 4.72
Income More Than Expenses	160	22.8	54.14 ± 14.34	48.31 ± 13.85	13.36 ± 4.54
Test and p			*F* = 1.747 *p* = 0.175	*F* = 4.753 ***p*** **= 0.009**	*F* = 5.912 ***p*** **= 0.003**
Family Type					
Nuclear Family	535	76.3	54.87 ± 13.83	50.38 ± 14.61	14.33 ± 4.76
Extended Family	166	23.7	56.46 ± 13.70	51.78 ± 13.33	14.67 ± 4.44
Test and p			*t* = −1.299 *p* = 0.194	*t* = −1.100 *p* = 0.272	*t* = −0.841 *p* = 0.401

*n* Sample Size, *SD* Standart Deviation

### Differences in Scale Scores by Sociodemographic Variables

Gender-based comparisons indicated that girls had significantly higher post-traumatic stress levels (*t* = 3.508, *p* < 0.001) and lower quality of life (*t* = 4.223, *p* < 0.001) compared to boys (Table 1).

Significant differences were observed in loneliness scores according to maternal education level (*F* = 4.423, *p* = 0.002). Post-hoc LSD analyses revealed that adolescents whose mothers were illiterate (16.67 ± 6.03) reported higher loneliness than those whose mothers graduated from middle school (14.50 ± 6.62), high school (13.42 ± 4.46), or university (14.28 ± 5.27). Similarly, those with mothers who completed only primary (14.90 ± 4.46) or middle school (14.50 ± 6.62) reported greater loneliness than those with mothers who completed high school (13.42 ± 4.46).

Paternal occupation was significantly associated with both post-traumatic stress (*F* = 3.403, *p* = 0.017) and loneliness scores (*F* = 2.859, *p* = 0.036). Post-hoc comparisons showed that adolescents whose fathers were civil servants (52.74 ± 13.02) had lower PTSD scores than those whose fathers were farmers (57.54 ± 14.24), workers (56.84 ± 13.84), or self-employed (55.61 ± 14.27). Interestingly, adolescents whose fathers were farmers (15.89 ± 4.71) reported lower loneliness compared to those whose fathers were civil servants (14.08 ± 4.67), workers (14.23 ± 4.66), and self-employed (14.33 ± 4.66) (Table 1).

Income perception was significantly related to quality of life (*F* = 4.753, *p* = 0.009) and loneliness (*F* = 5.912, *p* = 0.003). Adolescents whose family income exceeded expenses (48.31 ± 13.85) reported better QoL than those whose income was less than (54.02 ± 14.63) or equal to expenses (50.90 ± 14.31). Conversely, loneliness levels were higher among adolescents reporting income less than (15.21 ± 4.52) or equal to expenses (14.62 ± 4.72), compared to those reporting income greater than expenses (13.36 ± 4.54) (Table 1).

### Descriptive Statistics of Study Scales

The mean Post-Traumatic Stress Reaction Scale score was 55.25 ± 13.80 (range: 20–95, indicating severe PTSD), the mean Quality of Life score was 50.72 ± 13.80 (range: 23–113), and the mean UCLA Loneliness score was 14.41 ± 4.69 (range: 7–28) (Table 2).

**Table 2:** Adolescents’ PTSD-I, PedsQL and UCLA ULS-8 Scores and Cronbach Alpha Values

						Confidence Interval		
	n	Mean	SD	Min	Max	Lower	Upper	Alpha
Score of PTSD-I	701	55.25	13.80	20	95	54.22	56.27	.860
Score of PedsQL	701	50.72	14.32	23	113	49.65	51.78	.888
Score of ULS-8	701	14.41	4.69	7.0	28.0	14.06	14.76	.738

*SD* Standart Deviasyon, *Min* Minimum, *Max* Maximum

### Correlation Analyses

After applying the standard PedsQL reverse-scoring procedure (0–100 transformation), Pearson correlation analysis revealed that higher post-traumatic stress symptoms were significantly associated with lower quality of life (*r* = −0.474, *p* < 0.001). This moderate negative relationship aligns with the theoretical expectations and previous literature, indicating that increased PTSD symptoms correspond to poorer well-being. Weak negative correlations were also observed between PTSD and the UCLA Loneliness Scale (*r* = 0.215, *p* < 0.001), and between PedsQL and loneliness (*r* = −0.284, *p* < 0.001) (Table 3).

**Table 3:** Correlation Between Adolescents’ PTSD-I, PedsQL, and ULS-8 Scores

	PTSD-I	PedsQL	ULS-8
Child post-traumatic stress reaction index (PTSD-I)	1	*r* = −0.474**	*r* = 0.215**
		***p*** **< 0.001**	***p*** **< 0.001**
Pediatric Quality of Life Inventory (PedsQL)	*r* = −0.474**	1	*r* = −0.284**
	***p*** **< 0.001**		***p*** **< 0.001**
UCLA Loneliness Scale (ULS-8)	r = r = 0.215**	*r* = −0.284**	1
	***p*** **< 0.001**	***p*** **< 0.001**	

## Discussion

In addition to the material damage caused by earthquakes, they also cause great moral damage and remain in the memory of society for many years. It is clear that the trauma and psychological disorders that occur after earthquakes will have a greater impact on children than adults. In a study conducted, it was emphasized that children were the group most affected by earthquakes and trauma, hopelessness, anxiety, depression and post-traumatic stress disorder were detected in children after the earthquake (Gürbüz & Koyuncu, 2023). The findings obtained in this study, which was conducted to investigate the relationship between post-traumatic stress, quality of life and loneliness levels of adolescents after Kahramanmaraş-centered earthquakes, are discussed below.

### Demographic Characteristics and the Relationship Between Scales

In this study, adolescents’ demographic characteristics were associated with post-traumatic stress, quality of life (QoL), and loneliness. Female adolescents exhibited higher post-traumatic stress levels and lower QoL compared to males. This finding aligns with prior studies indicating that females tend to experience higher stress reactions and lower QoL after earthquakes (Zhou et al., 2020; Tang et al., 2017; Yılmaz et al., 2024; Chou et al., 2004, b; Liang et al., 2013; Tian et al., 2013). Social expectations for girls to show emotional sensitivity and concern for others may increase empathy and anxiety, while responsibility for family safety during disasters can elevate stress levels. In addition, biological factors such as hormonal fluctuations during adolescence, heightened hypothalamic–pituitary–adrenal (HPA) axis reactivity, and greater limbic system sensitivity to emotional stimuli have been shown to increase girls’ vulnerability to post-traumatic stress compared to boys.

Maternal education was significantly associated with adolescents’ loneliness. Adolescents whose mothers were illiterate or had lower education levels reported higher loneliness, consistent with studies showing that higher maternal education reduces depression risk and promotes positive psychological health in children (Huang et al., 2020; Padilla-Moledo et al., 2016). Maternal education likely improves family communication and emotional support, fostering security in adolescents and reducing feelings of loneliness.

Father’s occupation was significantly related to adolescents’ post-traumatic stress and loneliness scores. Although the literature lacks direct comparisons, it is plausible that paternal occupation influences family socioeconomic status, living conditions, and access to opportunities. Lower-income households may expose adolescents to more stressors, increasing post-traumatic stress and loneliness. Demanding occupations may reduce father-child interaction, weakening family support.

Perceived family income was significantly associated with QoL and loneliness. Adolescents who reported their family income as higher than expenses had better QoL and lower loneliness, whereas those with lower or equal income reported worse outcomes. Financial difficulties may reduce subjective well-being, restrict social opportunities, and increase loneliness (Gariépy et al., 2017; Hoyt et al., 2019; von Rueden et al., 2006), whereas higher income provides greater resources, social support, and psychologically healthier living conditions.

### Correlations among Post-Traumatic Stress, Quality of Life, and Loneliness

Moderate negative correlations were found between post-traumatic stress and QoL, and weak positive correlations with loneliness. These results indicate that as adolescents’ post-traumatic stress increases, QoL tends to decrease and loneliness increases. Longitudinal studies after major earthquakes, such as the Wenchuan earthquake in China and the Parnitha earthquake in Greece, highlight that trauma can reduce social engagement and QoL over time (Chen et al., 2023; Goenjian et al., 2011). Lack of social support and disrupted living conditions further exacerbate stress and loneliness (Sarman, & ve Tuncay, S., 2024; Ünver & Fiş, 2019; Yanardağ, 2024).

Additionally, a weak negative correlation was observed between QoL and loneliness, suggesting that decreased QoL may increase feelings of loneliness. Adolescents displaced to collective living environments, such as container cities after the Maraş earthquake, experienced restricted personal space, reduced opportunities for social interaction, and increased isolation (Yanardağ, 2024). These findings are consistent with previous research showing that natural disaster survivors face family losses, changes in living conditions, disrupted peer relationships, parental pressures, economic losses, nostalgia, and concerns about the future, all of which contribute to loneliness and reduced QoL (Alipour & Ahmadi, 2020; Marangöz & İzci, 2023).

Mental health problems are common among adolescents following natural disasters and vary depending on the type of disaster, frequency of exposure, and individual or familial factors. Depression, anxiety, and post-traumatic stress disorder (PTSD) are the most frequently observed conditions, with earthquakes and pandemics in particular increasing the risk of PTSD. Studies have shown that adolescents experience depression, anxiety, and behavioral problems after floods and storms, and that repeated exposures further elevate these risks (Auchincloss et al., 2024; Campbell et al., 2024). Other research has reported that wildfires are associated with PTSD, depression, anxiety, and fear among adolescents (Auchincloss et al., 2024; Mellado, 2024). In addition, adolescents exposed to multiple disasters have been found to experience behavioral and emotional problems more frequently (Campbell et al., 2024; Edwards et al., 2023). These findings underscore the inevitable need for long-term, multidimensional mental health services in the aftermath of disasters.

## Conclusion and Recommendations

The findings of the study show that posttraumatic stress levels and quality of life of adolescents are related to socio-demographic factors such as gender, mother’s education level, father’s occupation and income perception. Female adolescents were found to have higher levels of posttraumatic stress and lower quality of life compared to male adolescents. In addition, mother’s education level, father’s occupation and perception of income status were found to have a significant effect on adolescents’ loneliness levels and posttraumatic stress responses. These results emphasize the importance of considering socio-economic factors when determining the psychological and social support needs of adolescents. In the future, it is recommended that psychological support and guidance services to reduce posttraumatic stress and loneliness levels of adolescents should be provided in a more targeted manner, especially for groups at risk. In addition, improving the education and income status of parents may have a significant impact on improving the quality of life of adolescents.

## Limitations of the Study

The results obtained from the study can be generalized to adolescents studying in related schools.
